# Radiomics Analysis for Multiple Myeloma: A Systematic Review with Radiomics Quality Scoring

**DOI:** 10.3390/diagnostics13122021

**Published:** 2023-06-10

**Authors:** Michail E. Klontzas, Matthaios Triantafyllou, Dimitrios Leventis, Emmanouil Koltsakis, Georgios Kalarakis, Antonios Tzortzakakis, Apostolos H. Karantanas

**Affiliations:** 1 Department of Medical Imaging, University Hospital of Heraklion, 71110 Heraklion, Greece; manthostr@gmail.com (M.T.); dimitrios_leventis@yahoo.com (D.L.); 2 Department of Radiology, School of Medicine, University of Crete, Voutes Campus, 71003 Heraklion, Greece; 3 Department of Radiology, Karolinska University Hospital, 14152 Stockholm, Sweden; emmanouilkolts@gmail.com (E.K.); georgios.kalarakis@ki.se (G.K.); 4 Division of Radiology, Department for Clinical Science, Intervention and Technology (CLINTEC), Karolinska Institutet, 14152 Stockholm, Sweden; antonios.tzortzakakis@ki.se; 5 Medical Radiation Physics and Nuclear Medicine, Section for Nuclear Medicine, Karolinska University Hospital, 14186 Huddinge, Stockholm, Sweden

**Keywords:** multiple myeloma, radiomics, machine learning, metastases, radiomics quality score

## Abstract

Multiple myeloma (MM) is one of the most common hematological malignancies affecting the bone marrow. Radiomics analysis has been employed in the literature in an attempt to evaluate the bone marrow of MM patients. This manuscript aimed to systematically review radiomics research on MM while employing a radiomics quality score (RQS) to accurately assess research quality in the field. A systematic search was performed on Web of Science, PubMed, and Scopus. The selected manuscripts were evaluated (data extraction and RQS scoring) by three independent readers (R1, R2, and R3) with experience in radiomics analysis. A total of 23 studies with 2682 patients were included, and the median RQS was 10 for R1 (IQR 5.5–12) and R3 (IQR 8.3–12) and 11 (IQR 7.5–12.5) for R2. RQS was not significantly correlated with any of the assessed bibliometric data (impact factor, quartile, year of publication, and imaging modality) (*p* > 0.05). Our results demonstrated the low quality of published radiomics research in MM, similarly to other fields of radiomics research, highlighting the need to tighten publication standards.

## 1. Introduction

Radiomics represents the image-based equivalent of biological omics analyses (e.g., transcriptomics and proteomics), which promises to offer high-fidelity analysis of images for precision medicine purposes [1]. Analysis of regions of interest in medical images can be performed by extracting radiomics features, which can be used to construct machine learning models that achieve a precise diagnosis, treatment response prediction, and disease prognosis [2,3]. Radiomics has been introduced as a promising image analysis method equivalent to other omics analyses aiming to achieve an image-based biopsy of regions of interest. Nonetheless, radiomics has yet to reach clinical practice but represents a promising research tool for developing predictive image-based signatures that can assist in the diagnosis and treatment of various diseases [4,5]. The quality of radiomics studies can be assessed with the use of the radiomics quality score (RQS) [6,7], which scores radiomics research against a series of standards, including but not limited to the quality of reporting, segmentation, feature extraction, feature selection, the calibration and validation of machine learning models, and the provision of open access data.

Multiple myeloma (MM) is one of the most common hematological malignancies, characterized by osteolytic lesions [8]. The heterogeneous nature of the disease complicates its diagnosis and treatment, and the similarities of MM lesions relative to osteolytic metastases during imaging complicate the image-based diagnosis of the disease [9]. The diagnostic approach of MM is based on whole-body CT, MRI, and PET-CT [10]. A series of radiomics research papers utilizing images from these modalities have been published in an attempt to achieve the differentiation of MM from metastases, prediction of treatment response, identification of molecular subgroups of MM, and analysis of patient survival.

The aims of this systematic review were (a) to provide a comprehensive analysis of the applications of radiomics in MM research and (b) to score manuscripts using RQS to benchmark their quality against existing standards.

## 2. Materials and Methods

The protocol of this study has been registered with the PROSPERO international register for systematic reviews (https://www.crd.york.ac.uk/prospero/, Record ID CRD42023409189, accessed 8 June 2023). Preferred Reporting Items for Systematic Reviews and Meta-analyses (PRISMA) guidelines were used to prepare this manuscript [11].

### 2.1. Database Search Strategy and Selection of Relevant Studies

The search for relevant papers was performed between 1 January 2010 and 1 April 2023 in three databases (dPubMed, Scopus, and Web of Science). The search was performed with the strings “multiple myeloma”, “radiomics”, and “texture”. The detailed strings used in each of the three databases can be found in Supplementary File S1. Studies from all three databases were collected, and duplicate studies were excluded from further analysis. Three radiologists (MEK, MT, and DL) with 5, 2, and 2 years of experience in radiomics research examined the abstracts of all studies to exclude those that were not eligible: (1) review papers, (2) abstracts and conference papers, (3) editorials, (4) non-English papers. The records were further screened to exclude manuscripts that did not perform radiomics analysis.

### 2.2. Literature Data Extraction

From each included study, specific information was extracted, including author names, country of origin, year of publication, journal, number of patients, the purpose of the study, imaging modality used, software for radiomics analysis, and the type of MM. Each journal’s impact factor was recorded according to the 2021 Journal Citation Reports (Clarivate) and the journal’s quartile according to Scimago Journal and Country Rank (https://www.scimagojr.com, accessed 20 April 2023).

### 2.3. Radiomics Quality Score (RQS)

The quality of radiomics research presented in each study was evaluated by three readers blinded to the results of each other using RQS [6,7]. The first and most experienced reader (R1–MEK) had 5 years of experience in radiomics research and 10 years of experience in medical imaging research, whereas the second and third (R2 and R3—MT and DL) had 2 years of experience in radiomics and 3 years of experience in medical imaging research. Before the initiation of scoring, a training session was performed using radiomics papers unrelated to MM to ensure that manuscripts were scored in precisely the same manner by all readers. Each reader was blinded to the scores that the rest of the readers gave. The RQS consists of 16 items, with a total score ranging between −8 and 36. A total percentage (out of 36) was also calculated for each manuscript. 

### 2.4. Statistical Analysis

Statistical analysis was performed using SPSS v 29 (IBM SPSS for Mac, Armonk, NY, USA). Variables are expressed as frequencies and percentages (categorical) or medians with interquartile ranges (continuous). An adherence metric was calculated to assess adherence to RQS by awarding one point if the authors had gained the minimum points for each RQS item. Data normality was assessed using the Shapiro–Wilk test. RQS comparisons between groups of studies with different characteristics (impact factor, year of publication, journal quartile, and modality) were performed using Mann–Whitney U or Kruskall–Wallis tests according to the number of groups. Agreement between readers was evaluated using the intraclass correlation coefficient (ICC) assessed for absolute agreement, considering acceptable ICC with values >0.75 (good: 0.75–0.9, excellent > 0.9). Statistical significance was defined with α set at 0.05.

## 3. Results

### 3.1. Study Selection

**Figure 1:**
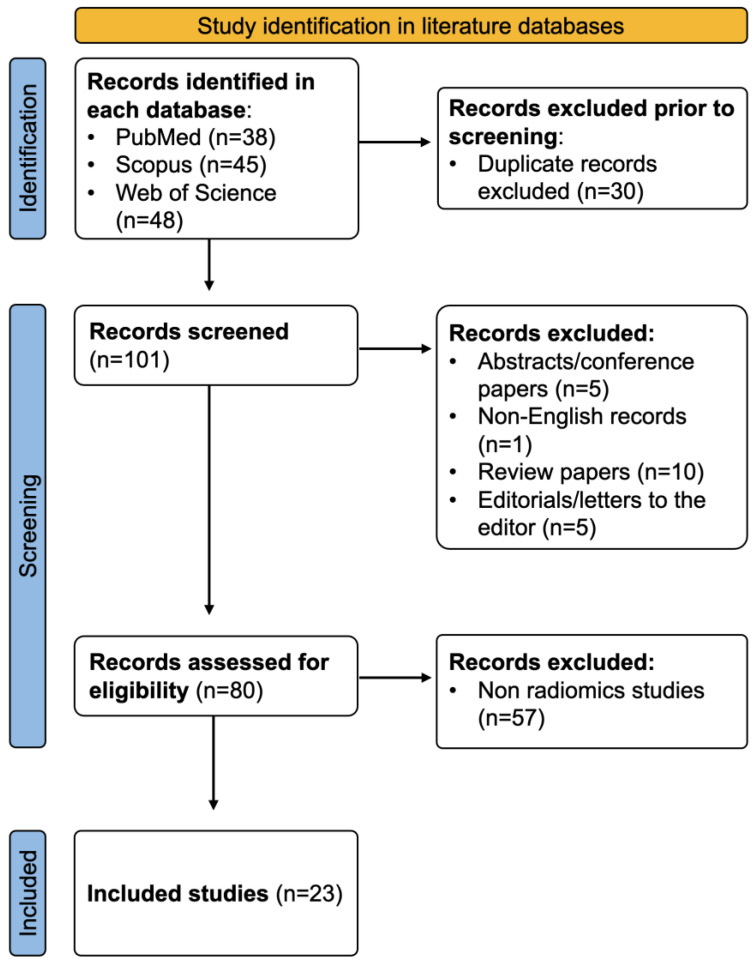
Flow chart detailing study selection methodology.

After excluding duplicate entries (n = 30), 101 records from all three databases were screened. A total of 21 studies were excluded due to being an inappropriate manuscript type (non-English, review, conference paper, and editorial/letter to the editor), and 57 studies were excluded because of a non-radiomics study design (studies that did not extract radiomics features). This yielded a final sum of n = 23 manuscripts, which were included for analysis (Figure 1). 

### 3.2. Analysis of Included Studies

Detailed characteristics of the 23 included studies can be found in Table 1. In total, 8 out of 23 studies (34.8%) were published in radiology journals, 4/23 (17.4%) were published in oncological journals, 3/23 (13%) were published in nuclear medicine journals. More than half of the studies (12/23) were published in journals with an Impact Factor > 5. Most studies were published in Q1 journals (15/23—65.2%), with five studies in Q2 (21.7%), two studies in Q3, and one study in a Q4 journal. No study was published before 2019, whereas nine studies were published in each of the years 2021 and 2022. China and Germany were the main countries of origin for published studies, with 34.8% and 21.7% of the studies originating from each of the two, respectively. 

The methodological details of individual studies are presented in Table 2. A total of 2682 patients were examined in the 23 included studies. Several radiomics applications on multiple myeloma have been described in the studies included, with the majority (6 out of 23 studies—26.1%) aimed at differentiating between MM and metastases and the same proportion aimed at predicting treatment response or prognosis. Only 4/23 studies aimed at diagnosing MM compared to normal bone marrow or other hematological conditions. Three out of twenty-three studies evaluated the pattern of infiltration of the bone marrow. In contrast, other miscellaneous applications such as the disease’s load prediction and the presence of high-risk cytogenetic abnormalities represented the minority of the literature (1–2 studies for each application). One of the studies aimed at evaluating the technical reproducibility of radiomics in patients with MM. Almost half of the studies utilized MRI (11/23—47.8%), with PET-CT and CT (single or dual-energy) being the second-most common modalities (6/23 studies each—26.1%). Various commercial and free software was used; a detailed list can be found in Table 2. Finally, most studies did not define which specific MM type was examined.

### 3.3. Radiomics Quality Score (RQS)

**Figure 2:**
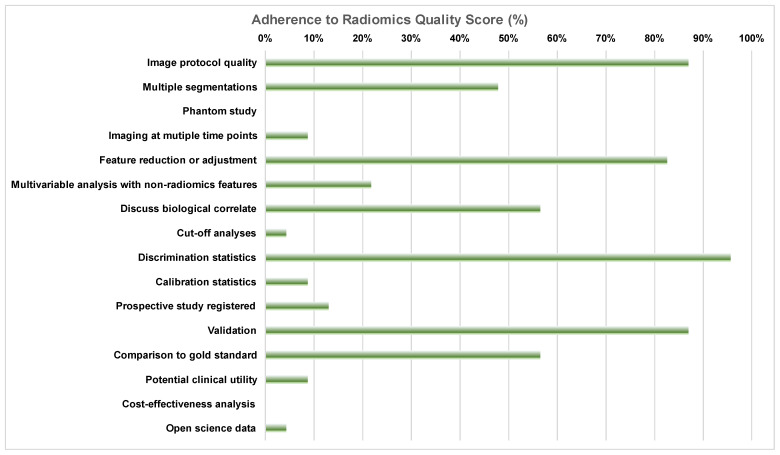
Adherence to radiomics quality score items according to the most experienced reader (R1), expressed as the percentage of manuscripts that received the minimum score for each individual item.

Adherence to RQS items varied significantly. As shown in Figure 2, 96% of manuscripts contained some discrimination statistics (e.g., AUC with 95% confidence intervals), and more than 85% (87%) of papers included a sufficiently detailed imaging protocol. It is important that 13% of the studies did not attempt any validation (including internal testing). In contrast, only 9% of the studies included calibration statistics for the presented machine learning models. Feature reduction to account for the possibility of overfitting was performed in 83% of manuscripts, and 57% of studies established the ground truth with the use of a gold standard (e.g., bone marrow biopsy). Finally, only 4% of manuscripts provided either an open access code or open access data, whereas no manuscript included a cost-effectiveness analysis or phantom standardization of radiomics features.

**Figure 3:**
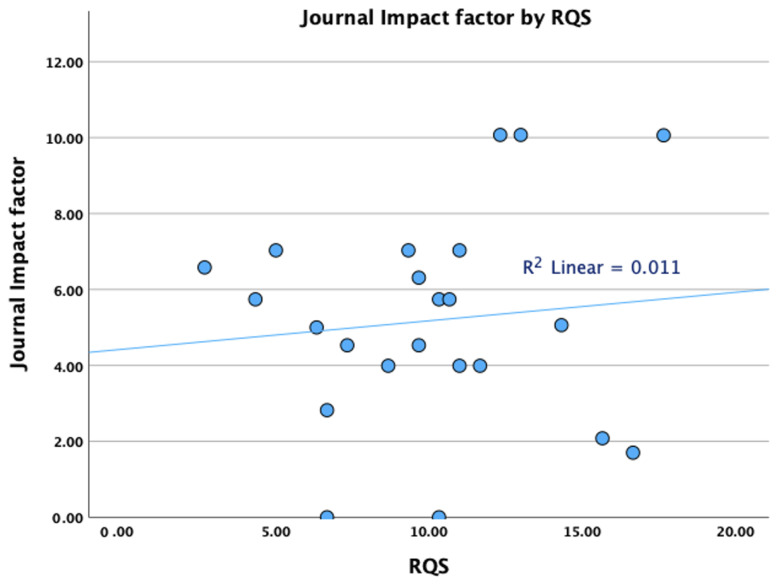
Scatter plot demonstrating the relationship between journal impact factors and radiomics quality scores.

The total RQS for each of the studies for all three readers is presented in Table 3. The median RQS was 10 for R1 (IQR 5.5–12) and R3 (IQR 8.3–12) and 11 (IQR 7.5–12.5) for R2. The agreement between readers was good (almost excellent), with an ICC of 0.851 (95% CI 0.7 to 0.932). Subgroup analysis showed that there are no statistically significant differences between RQS for papers published in journals with a high (>5) and low impact factor (Figure 3 and Table 4), between papers published before or after 2021, or papers in high or low quartiles or papers dealing with different imaging modalities (Table 4). Impact factor analysis was repeated by removing the only methodological study (which does not contain a predictive model) from our group [32], noting that there was still no significant correlation between the impact factor and the RQS score (*p* > 0.05 for all readers).

## 4. Discussion

Herein, we presented a comprehensive analysis of radiomics studies on MM. We analyzed study characteristics and demonstrated that the quality of radiomics studies published for the evaluation of MM is inadequate. Importantly, we demonstrated low adherence rates to most RQS items and a low total RQS, and we showed that low quality is generic and not specific to the journal’s characteristics. This finding is extremely important in evaluating published research and highlights the need to conduct high-quality research in the field.

The issue of inadequate-quality published radiomics research has been recently highlighted [5] in studies dealing with various topics [35,36], strongly indicating a potential lack of reproducibility. Most examined studies did not include external validation of their results, with two of them also skipping testing using internal data. The lack of external validation significantly limits the generalization capacity of machine learning models and is a common problem in published papers on AI using medical images, where only 6–10% of published studies have been tested on an external dataset [37]. This is of high importance since algorithm performance is consistently lower when validated on external datasets and even lower in “real-life” conditions [37,38]. Our findings signify the fact that even though the published algorithms may have the potential to revolutionize MM diagnosis and management, the lack of external validation hinders their adoption, reducing the trust in the results.

RQS is, at the moment, the most important tool for evaluating radiomics research. Adherence to RQS items is supposed to indicate high-quality research. In our case, some items were either addressed in none or a very limited subset of the studies, including the use of phantoms, cost-effectiveness analysis, and the provision of open access data. The lack of open access data may be attributed to limitations related to the publication of patient data. However, anonymized radiomics numerical values do not fall under this category. The publication of such studies indicates a lack of robust reviewing practices and insufficient journal and reviewer expertise in radiomics and machine learning. The rapid increase in machine learning publications in musculoskeletal imaging [39] has caused an increased demand for expert reviewers. Thus, assigning such manuscripts to inexperienced reviewers with potential expertise in musculoskeletal or hematological malignancies can lead to the publication of low-quality research, even in high-impact journals. As indicated by our results, there was no difference between high- and low-impact journals regarding their RQS score. To overcome this problem, guides for radiomics research have been published [1,3], providing basic directions to reviewers; moreover, journals need to adhere to the basic standards of RQS. It also needs to be pointed out that RQS has not been specifically designed to evaluate studies that do not contain predictive models. Therefore, technical radiomics studies may receive a lower RQS score because they present no predictive models. Our study included one such technical manuscript by Wennmann et al. [31], which received a median RQS of 12. This is almost identical to the median RQS of all studies in our sample; therefore, it was not analyzed separately since it does not negatively affect the results of the study. However, it needs to be pointed out that such studies can be of higher quality than the quality indicated by their RQS score since they are negatively scored for the absence of a predictive model that they were not supposed to analyze. 

Radiomics, a rapidly evolving field within medical imaging, focuses on extracting high-dimensional quantitative data from medical images, utilizing these data to uncover hidden information that is not readily discernible by the human eye [1]. This innovative approach presents a promising frontier for diagnosing MM and the differentiation between MM and lytic metastases. The complex nature of MM lesions, coupled with their variable appearance in imaging, creates a pressing need for advanced diagnostic techniques that enable accurate, efficient, and personalized interventions for affected patients. MM is a hematologic malignancy characterized by lytic bone lesions, making their distinction from lytic metastases caused by other types of malignancy a challenging task for clinicians [10]. Accurate diagnosis and differentiation are crucial as the treatment strategies, prognoses, and overall management of MM patients are markedly different from those with metastatic bone disease. Radiomics, by leveraging advanced machine learning algorithms and computational models, has the potential to identify unique, subvisual patterns and features within MM-affected bones that can accurately distinguish them from lytic metastases. Radiomics features provide a comprehensive, quantitative analysis of tumor characteristics, which can improve diagnostic accuracy and potentially reveal novel imaging biomarkers for MM [3,4]. Consequently, these advancements may lead to earlier and more personalized interventions for patients with multiple myeloma, ultimately enhancing their prognosis and quality of life. 

One of the most important reasons that radiomics has not yet found extensive applications on MM is the nature of MM lesions, which are disseminated across the bone marrow, presenting an important challenge with regard to the segmentation of the entire tumor load given that this is not evenly distributed across the skeleton [40] and the mutations associated with the lesions can be spatially heterogeneous [41]. Therefore, in order to implement radiomics in clinical practice for MM patients, methods that allow the segmentation of multiple focal lesions and extended areas of diffuse infiltration or whole bones are required since the manual segmentation of this scale is extremely tedious and potentially unreliable. Methods such as the atlas-based semi-automatic segmentation of whole-body diffusion-weighted imaging and deep learning applications combined with radiomics have already been proposed [31,42,43,44] and may be the solution to the future translation of radiomics research to the clinic.

Several medical imaging modalities, including computed tomography (CT), magnetic resonance imaging (MRI), and positron emission tomography (PET), can be utilized for radiomic analysis in MM patients. Our work showed that almost half of the manuscripts utilized MRI-based radiomics. CT-derived radiomics features may capture structural alterations in the trabecular bone, while MRI-based radiomics can offer insights into the tissue composition in the bone marrow. PET-based radiomics, on the other hand, can evaluate the metabolic activity of MM lesions and assess their response to therapy [8,10]. By integrating multi-modal imaging data, radiomics offers a comprehensive view of MM lesions, potentially enabling more accurate and precise diagnoses. The future integration of radiomics with other omics data, such as genomics, proteomics, and metabolomics, could create a holistic understanding of MM, uncovering complex relationships between imaging phenotypes and the underlying molecular mechanisms driving disease progression. This integration, often referred to as radiogenomics or radiotranscriptomics [45,46], can pave the way for personalized medicine approaches in MM, guiding clinicians in tailoring treatment plans based on the specific characteristics of each patient’s disease. Utilizing such integration methods for the diagnosis of MM would necessitate high-quality radiomics methods that are not currently available in the MM literature. The development of high-quality radiomics signatures for implementation in the clinical management of multiple myeloma requires interdisciplinary collaboration among radiologists, oncologists, physicists, data scientists, and bioinformaticians. This collaborative approach can foster the development of novel radiomic models, drive the discovery of new imaging biomarkers, and ultimately contribute to the improvement of diagnostic accuracy, treatment planning, and overall patient outcomes.

Our study has certain strengths and limitations. The systematic review of the literature and the comprehensive analysis of study characteristics represent the strengths of our study. Another important strength is the high ICC between the three readers. Such high agreement between readers has been reported in other RQS assessment studies [47]. Nonetheless, one limitation of our study is related to the inherent limitations of RQS. The score itself is empirical and was not validated when first published by Lambin et al. [4]. Therefore, it includes items, such as the use of phantoms for feature standardization and cost-effectiveness analysis, that are consistently not found in any published radiomics papers, even the ones that adhere to the highest quality standards [36,47,48]. Thus, it has been suggested that these items could unnecessarily reduce the total RQS, preventing studies from obtaining the maximum number (36) of points. Nonetheless, in our study, the maximum total RQS was 20, which cannot be compensated by scoring points in the items mentioned above. In similar RQS systematic reviews for other types of disease, the maximum RQS score was similar. The same is true for the median RQS value, which was found to be 10 in our study, comparable to values shown by other published systematic reviews. Such examples include a maximum of 22 in studies of hepatocellular carcinoma [49] and a maximum of 16 in studies of ischemic strokes [50].

## 5. Conclusions

In conclusion, radiomics presents a powerful and innovative approach for evaluating MM. By unlocking the hidden information within medical images, radiomics has the potential to revolutionize MM diagnosis, risk stratification, and treatment planning. Our study has highlighted the low scientific quality of radiomics papers related to MM, similarly to other fields of radiomics research, demonstrating that the methodological limitations of existing studies are not related to bibliometric (journal, impact factor, quartile, etc.) data. These findings emphasize the need to tighten current publication standards in order to publish radiomics research studies of high quality.

## Figures and Tables

**Table 1:** Study characteristics.

Study	Journal	Journal Field	Journal Quartile	JCR Impact Factor (2021)	Year	Country
Chen [12]	*Frontiers in Oncology*	Oncology	Q1	5.738	2022	China
Ekert [13]	*Cancers*	Oncology	Q1	6.575	2020	Germany
Hwang [14]	*Scientific Reports*	General	Q1	4.996	2019	Korea
Jamet [15]	*European Journal of Nuclear Medicine and Molecular Imaging*	Nuclear Medicine	Q1	10.057	2021	France
Jin [16]	*Frontiers in Medicine*	General	Q1	5.058	2022	China
Lee [17]	*Diagnostics*	General	Q2	3.992	2023	Korea/USA
Li [18]	*Frontiers in Oncology*	Oncology	Q1	5.738	2021	China
Li [19]	*BioMed Research International*	General	Q2	NA	2022	China
Liu [20]	*European Radiology*	Radiology	Q1	7.034	2021	China
Liu [21]	*Radiologia Medica*	Radiology	Q1	6.313	2021	China
Mannam [22]	*Indian Journal of Nuclear Medicine*	Nuclear Medicine	Q4	NA	2022	India
Mesguich [23]	*Nuclear Medicine Communications*	Nuclear Medicine	Q3	1.698	2021	France
Milara [24]	*Computer Methods and Programs in Biomedicine*	Computer Science	Q1	7.027	2022	Spain
Park [25]	*Diagnostics*	General	Q2	3.992	2022	Korea
Reinert [26]	*European Radiology*	Radiology	Q1	7.034	2020	Germany
Reinert [27]	*European Journal of Radiology*	Radiology	Q1	4.531	2021	Germany
Ripani [28]	*Clinical Lymphoma, Myeloma and Leukemia*	Hematology	Q2	2.822	2021	Italy
Schenone [29]	*Diagnostics*	General	Q2	3.992	2021	Italy
Tagliafico [30]	*European Journal of Radiology*	Radiology	Q1	4.531	2022	Italy
Wennmann [31]	*Investigative Radiology*	Radiology	Q1	10.065	2022	Germany
Wennmann [32]	*Investigative Radiology*	Radiology	Q1	10.065	2023	Germany
Wu [33]	*Journal of Computer Assisted Tomography*	Radiology	Q3	2.081	2022	China
Xiong [34]	*Frontiers in Oncology*	Oncology	Q1	5.738	2021	China

**Table 2:** Methodological characteristics of included studies.

Study	Patients (Training/Validation)	Number of Extracted Features (Location of ROI/VOI)	Purpose	Modality	Sequences Used	Software Used (Segmentation/Feature Extraction/Model Building	MM Type/Specific Location
Chen [12]	217 (No test set, but 5-fold CV)	30 (spinal focal bone lesions)	Differential diagnosis between MM and metastases	MRI	CE T1-w	ITK-SNAP/python	Spinal MM
Ekert [13]	67	92 (focal bone marrow lesions)	Prediction of treatment response	MRI	WB T1-w, ADC, STIR	Not Specified	Not Specified
Hwang [14]	467 (360/107)	56,862 (whole lumbar spine bone marrow)	Differential diagnosis between hematological malignancies and normal marrow	MRI	T1-w	MATLAB	Not Specified
Jamet [15]	139 (98/41)	17 (focal bone lesions and extramedullary disease)	Prediction of Prognosis	PET-CT		Python	Not Specified
Jin [16]	131 (70:30 split)	279 (spinal lesions)	Differential diagnosis between MM and metastases	PET-CT		MadZa/python	Spinal MM
Lee [17]	225 (175/50)	1218 (focal bone lesions)	Differential diagnosis between MM and metastases	CE CT		ITK-SNAP, mlr package	Not Specified
Li Y [18]	121 (84/37)	1316 (whole lumbar spine bone marrow)	Prediction of Prognosis	MRI	T1-w, T2FS	ITK_SNAP, R programming	Spinal MM
Li Y [19]	95 (77 /18)	1316 (whole lumbar spine bone marrow)	Prediction of treatment response	MRI	T1-w, T2FS	AI Kit software AK GE, ITK-SNAP, R programming	Not Specified
Liu [20]	241 (80:20 split)	1409 (spinal focal bone lesions)	Differential diagnosis between MM and metastases	MRI	T1-w, T2-w FS	RadCloud AI Scientific Research platform	Spinal MM
Liu [21]	50 (4/5 of patients in training set)	1409 (spinal focal bone lesions)	Prediction of high risk Cytogenetic abnormalities	MRI	T1-w, T2-w, T2FS	RadCloud platform	Spinal MM
Mannam [22]	40 (26/14)	138 (focal bone lesions)	Differential diagnosis between MM and metastases	PET-CT	CT venous phase	LifeX/Weka Data Mining/XLSTAT	Not Specified
Mesguich [23]	30 (20/10)	174 (whole spinal bone marrow)	Diagnosis of MM (no alternative diagnosis)	PET-CT	CT and PET SUV	Horos/Python	Not Specified
Milara [24]	39 (no test set, only 5-fold cross validation)	27 (whole skeleton)	Prediction of disease load	PET-CT		MATLAB/Statical Parametric Mapping 12/Orange	Not Specified
Park [25]	208 (160/48)	1218 (whole axial skeleton)	Diagnosis of MM (no alternative diagnosis)	CE-CT		Syngo.via/R programming	Not Specified
Reinert [26]	110 (no validation)	92 (bone marrow of vertebral bodies T10-L5)	Prediction of bone marrow infiltration	DE-CT		Syngo.via/Python	Spinal MM
Reinert [27]	44	41 (whole bone marrow)	Correlation with hematological parameters	DE-CT		Syngo.via	Not Specified
Ripani [28]	45	Unspecified (whole bone marrow)	Prediction of disease progression	PET-CT		LifeX	Smoldering MM
Schenone [29]	33	109 (focal bone lesions)	Prediction of treatment response	CT		Bone GUI, 3D Slicer	Not Specified
Tagliafico [30]	60	Not specified (paraspinal muscle sarcopenia assessment)	Prediction of MM phenotype (focal vs. diffuse)	CT		3D Slicer	Not Specified
Wennmann [31]	102 (50/52)	296 (“bone marrow spaces”)	Prediction of MM phenotype (normal vs. focal vs. diffuse)	MRI	WB T1-w	Python	Smoldering MM
Wennmann [32]	55	295 (bone marrow of the left hip)	Assessment of radiomics reproducibility	MRI	T1-w, T2-w	MITK phenotyping/Python	Not Specified
Wu [33]	123 (85/38)	1702 (focal bone lesions)	Prediction of treatment response	MRI	T1-w, T2-w, STIR	3D Slicer/R programming	Spinal MM
Xiong [34]	107 (75/32)	282 (lumbar focal bone lesions)	Differential Diagnosis between MM and metastases	MRI	T1-w, T2-w	MadZa/R programming	Spinal MM

**Table 3:** Total radiomics quality score for each study per reader.

Study	RQS of R1	RQS of R2	RQS of R3
Chen [12]	5	5	3
Ekert [13]	3	2	3
Hwang [14]	5	5	9
Jamet [15]	15	18	20
Jin [16]	13	14	16
Lee [17]	12	13	10
Li Y [18]	9	12	10
Li Y [19]	9	11	11
Liu [20]	12	11	10
Liu [21]	10	12	7
Mannam [22]	0	10	10
Mesguich [23]	15	15	20
Milara [24]	10	10	8
Park [25]	11	11	11
Reinert [26]	3	3	9
Reinert [27]	6	6	10
Ripani [28]	3	3	14
Schenone [29]	9	9	8
Tagliafico [30]	9	9	11
Wennmann [31]	12	12	13
Wennmann [32]	15	15	9
Wu [33]	17	17	13
Xiong [34]	12	12	8

**Table 4:** Factor subgroup analysis of radiomics quality scores for each reader. Numbers represent median scores (interquartile range).

Category	*N*	Reader 1		Reader 2		Reader 3	
Journal Quartile			*p = 0.686*		*p = 0.399*		*p = 0.241*
Q1	15	10 (5.5–12)		11 (5.5–12)		9 (8–10.5)	
Q2	5	9 (9–11)		11 (9–11)		11 (10–11)	
Q3–4	3	15 (7.5–16)		15 (12.5–16)		13 (11.5–16.5)	
Impact Factor			*p = 0.68*		*p = 0.214*		*p = 0.214*
1–5	11	9 (5.5–11.5)		10 (7.5–12)		11 (10–12)	
>5	12	11 (8–12.25)		12 (8.8–12.5)		9 (7.8–10.8)	
Modality			*p = 0.631*		*p = 0.432*		*p = 0.393*
CT	3	8.5 (5.3–11.3)		8.5 (8–12)		10 (7.5–10.5)	
MRI	11	10 (7–12)		12 (8–12)		9 (7.5–10.5)	
PET-CT	6	9.5 (7.5–13.5)		10 (9–14.3)		12.5 (9.5–17)	
Publication Year			*p = 0.414*		*p = 1*		*p = 0.214*
2010–2021	12	9 (4.5–12)		10 (4.5–12)		9.5 (8–11)	
2022–2023	11	11 (9–12.5)		11 (10–13.5)		11 (9.5–12)	

## Data Availability

All data required to reproduce results are provided in the paper.

## Supplementary Materials

The following supporting information can be downloaded at https://www.mdpi.com/article/10.3390/diagnostics13122021/s1. Supplementary File S1: Full strings used for database search.
