# Supplementary material for: Radiomics Analysis for Multiple Myeloma: A Systematic Review with Radiomics Quality Scoring
Source: Diagnostics (Basel). 2023 Jun 10;13(12):2021. doi: 10.3390/diagnostics13122021 (PMC10296889; doi:10.3390/diagnostics13122021)
Supplement: Supplementary file 1 [file diagnostics-13-02021-s001.zip › diagnostics-2368862-supplementary.pdf]

PUBMED

((((texture) OR (radiomics))) AND ((multiple myeloma))) AND (("2010/01/01"[Date - Publication] : "2023/04/01"[Date - Publication]))

Scopus

(TITLE-ABS-KEY (radiomics) OR TITLE-ABS-KEY (texture) AND TITLE-ABS-KEY (multiple AND myeloma)) AND PUBYEAR > 2009

Web of science

(((ALL=(texture)) OR ALL=(radiomics))) AND ALL=(multiple myeloma)

Publication date: 01-01-2010 to 01-04-2023
